# Simultaneous screening for COVID-19 and tuberculosis, India

**DOI:** 10.2471/BLT.22.288960

**Published:** 2023-05-18

**Authors:** Keertana Duppala, Rajashree Sen, Shubhada Shenai, Mangala Gomare, Daksha Shah, Pranita Tipre, Madhav Joshi, Jayeeta Chowdhury, Sarabjit S Chadha, Sanjay Sarin

**Affiliations:** a FIND, Flat No. 8, 9th floor, Vijaya Building, 17 Barakhamba Road, New Delhi 110001, India.; b Brihanmumbai Municipal Corporation, Mumbai, India.; c Confluence for Health Action and Transformation Foundation, Mumbai, India.

## Abstract

**Objective:**

To evaluate the implementation of new operational workflows for simultaneous screening of coronavirus disease 2019 (COVID-19) and tuberculosis at four high-volume COVID-19 testing centres located in tertiary hospitals in Mumbai, India.

**Methods:**

Each centre already offering antigen-detecting rapid diagnostic tests were equipped with a rapid molecular testing platform for COVID-19 and tuberculosis, sufficient laboratory staff, and reagents and consumables for screening. Using a verbal tuberculosis questionnaire, a patient follow-up agent screened individuals visiting the COVID-19 testing centres. Presumptive tuberculosis patients were asked to provide sputum samples for rapid molecular testing. Subsequently, we reversed our operational workflow to also screen patients visiting tuberculosis outpatient departments for COVID-19, using rapid diagnostic tests.

**Results:**

From March to December 2021, we screened 14 588 presumptive COVID-19 patients for tuberculosis, of whom 475 (3.3%) were identified as having presumptive tuberculosis. Of these, 288 (60.6%) were tested and 32 individuals (11.1%) were identified as tuberculosis positive (219 cases per 100 000 individuals screened). Of the tuberculosis-positive individuals, three had rifampicin-resistant tuberculosis. Among the remaining 187 presumptive tuberculosis cases not tested, 174 reported no symptoms at follow-up and 13 individuals either refused testing or could not be traced. Of the 671 presumptive tuberculosis cases screened for COVID-19, 17 (2.5%) were positive by antigen rapid diagnostic tests, and five (0.7%) who tested negative, later tested positive on the molecular testing platform (2483 COVID-19 cases per 100 000 individuals screened).

**Conclusion:**

Simultaneous screening for COVID-19 and tuberculosis in India is operationally feasible and can improve real-time on-site detection of COVID-19 and tuberculosis.

## Résumé

**Objectif:**

Évaluer la mise en œuvre de nouveaux processus opérationnels pour le dépistage simultané de la maladie à coronavirus 2019 (COVID-19) et de la tuberculose au sein de quatre grands centres de dépistage de la COVID-19 situés dans des hôpitaux tertiaires à Mumbai, en Inde.

**Méthodes:**

Une plateforme de tests moléculaires rapides pour la COVID-19 et la tuberculose, des agents de laboratoire en suffisance ainsi que des réactifs et consommables destinés au dépistage ont été mis à la disposition de chaque centre proposant déjà des tests de diagnostic rapide basés sur la détection d'antigènes. Par le biais d'un questionnaire oral sur la tuberculose, un agent chargé du suivi des patients a sélectionné des individus se rendant dans les centres de dépistage de la COVID-19. Les patients potentiellement atteints d'une tuberculose ont été invités à fournir des échantillons d'expectorations en vue de réaliser des tests moléculaires rapides. Ensuite, nous avons inversé notre processus opérationnel et utilisé des tests de diagnostic rapide afin de dépister la COVID-19 chez les patients ayant effectué une visite dans des cliniques externes dédiées à la tuberculose.

**Résultats:**

Entre mars et décembre 2021, 14 588 cas présumés de COVID-19 ont été retenus pour un dépistage de la tuberculose; 475 (3,3%) d'entre eux présentaient une suspicion de tuberculose. Au sein de cette catégorie, 288 (60,6%) individus ont été testés, dont 32 (11,1%) se sont révélés positifs à la tuberculose (219 cas pour 100 000 individus sélectionnés). Sur l'ensemble de ces individus positifs, trois souffraient d'une tuberculose résistant à la rifampicine. Parmi les 187 cas présumés de tuberculose restants qui n'ont pas été testés, 174 n'ont déclaré aucun symptôme lors du suivi et 13 ont refusé d'être testés ou n'ont pas été retrouvés. D'autre part, sur les 671 cas présumés de tuberculose soumis à un dépistage de la COVID-19, 17 (2,5%) ont obtenu un résultat positif lors des tests de diagnostic rapide basés sur la détection d'antigènes et cinq (0,7%) ont obtenu un résultat négatif, avant d'obtenir un résultat positif via la plateforme de tests moléculaires (2483 cas de COVID-19 pour 100 000 individus sélectionnés).

**Conclusion:**

Le dépistage simultané de la COVID-19 et de la tuberculose en Inde est non seulement réalisable sur le plan opérationnel, mais peut aussi améliorer la détection de la COVID-19 et de la tuberculose sur site et en temps réel.

## Resumen

**Objetivo:**

Evaluar la implementación de nuevos flujos de trabajo operativos para llevar a cabo exámenes de detección simultánea de la enfermedad de coronavirus 2019 (COVID-19) y de tuberculosis en cuatro centros de pruebas de alta incidencia de COVID-19, ubicados en hospitales terciarios de Mumbai (India).

**Métodos:**

Se equipó a cada uno de los centros que ya ofrecía pruebas de diagnóstico rápido de detección de antígenos con una plataforma de pruebas rápidas moleculares para detectar COVID-19 y tuberculosis. Asimismo, se les dotó de suficiente personal de laboratorio, de reactivos y de material para realizar los exámenes de detección. Mediante un cuestionario oral sobre tuberculosis, la persona responsable del seguimiento de pacientes evaluó a las personas que visitaban los centros de pruebas de COVID-19. Se solicitó a los pacientes que presuntamente padecían tuberculosis que proporcionaran muestras de esputo para realizar pruebas rápidas moleculares. Posteriormente, utilizando pruebas de diagnóstico rápido, se cambió el flujo de trabajo operativo con el fin de realizar también exámenes de detección de COVID-19 a pacientes que visitaban centros ambulatorios de tuberculosis.

**Resultados:**

De marzo a diciembre de 2021, se realizaron exámenes de detección de tuberculosis a 14 588 pacientes que presuntamente padecían COVID-19; entre estos pacientes, se identificó que 475 (3,3%) sufría una presunta tuberculosis. Entre estos pacientes, se realizaron pruebas a 288 (60,6%), llegándose a detectar tuberculosis positiva (219 casos por cada 100 000 individuos examinados) en 32 pacientes (11,1%). Entre los individuos con tuberculosis positiva, tres de ellos presentaban tuberculosis resistente a la rifampicina. De los 187 casos restantes no sometidos a pruebas y con presunta tuberculosis, 174 individuos informaron sobre la ausencia de síntomas en el seguimiento, y las 13 personas restantes o bien se negaron a hacerse las pruebas o no fue posible realizar su seguimiento. De los 671 casos con presunta tuberculosis en los que se llevó a cabo un examen de detección de COVID-19, 17 personas (2,5%) dieron positivo en pruebas de diagnóstico rápido de antígenos, y cinco (0,7%) que dieron negativo en un principio arrojaron posteriormente un resultado positivo en la plataforma de pruebas moleculares (2483 casos de COVID-19 por cada 100 000 individuos examinados).

**Conclusión:**

El examen de detección simultánea de COVID-19 y tuberculosis de la India es factible a nivel operativo y puede mejorar la detección de casos de COVID-19 y tuberculosis *in situ* en tiempo real.

## ملخص

**الغرض:**

تقييم تنفيذ تدفقات العمل التشغيلية الجديدة للفحص المتزامن لمرض فيروس كورونا 2019 (كوفيد 19) والسل، في أربعة مراكز عالية السعة لاختبار كوفيد 19 تقع في المستشفيات الثالثة في مومباي بالهند.

**الطريقة:**

كل مركز يقدم بالفعل اختبارات تشخيصية سريعة لاكتشاف المستضد، تم تجهيزه بمنصة اختبار جزيئي سريعة لكوفيد 19 والسل، وعدد كافٍ من فرق العمل في المختبرات، والكواشف والمواد الاستهلاكية للفحص. باستخدام الاستبيان الشفهي للسل، قام وكيل متابعة المريض بفحص الأفراد الذين يزورون مراكز اختبار كوفيد 19. طُلب من الأشخاص المفترض إصابتهم بالسل تقديم عينات من البلغم للاختبار الجزيئي السريع. بعد ذلك، قمنا بعكس مسار العمل التشغيلي لدينا أيضًا لفحص المرضى الذين يزورون أقسام العيادات الخارجية لمرض السل للكشف عن الإصابة بكوفيد 19، باستخدام اختبارات التشخيص السريع.

**النتائج:**

من مارس/آذار إلى ديسمبر/كانون أول 2021، قمنا بفحص 14588 مريضًا مفترضًا بكوفيد 19 للكشف عن الإصابة بمرض السل، تم تحديد أن 475 (3.3%) منهم يُفترض إصابتهم بالسل. من بين هؤلاء، تم اختبار 288 (60.6%)، وتم تحديد 32 فردًا (11.1%) على أنهم مصابون بمرض السل (219 حالة لكل 100000 فرد تم فحصهم). ومن بين الأفراد المصابين بالسل، كان ثلاثة منهم مصابين بالسل المقاوم للريفامبيسين. من بين الحالات المفترضة للإصابة بالسل الـ 187 المتبقية التي لم يتم اختبارها، أبلغت 174 حالة عن عدم وجود أعراض أثناء المتابعة، بينما هناك 13 شخصًا إما رفضوا الاختبار أو لا يمكن تتبعهم. من بين 671 حالة من الحالات المفترض إصابتها بالسل، التي تم فحصها للكشف عن الإصابة بكوفيد 19، كانت هناك 17 حالة (2.5%) إيجابية من خلال اختبارات التشخيص السريع للمستضد، وخمسة (0.7%) كانت نتيجة اختبارهم سلبية، ثم جاءت نتيجة اختبارهم لاحقًا إيجابية على منصة الاختبار الجزيئي (2483 حالة كوفيد 19 لكل 100000 فرد تم فحصهم).

**الاستنتاج:**

إن الفحص المتزامن لكل من كوفيد 19 والسل في الهند ممكن عمليًا، ويمكن أن يُحسّن الكشف في الوقت الفعلي في الموقع عن كوفيد 19 والسل.

## 摘要

**目的:**

评估在印度孟买三级医院的四个大型新型冠状病毒肺炎检测中心进行的新型冠状病毒肺炎 (COVID-19) 和结核病同步筛查工作的新操作流程的实施情况。

**方法:**

每个已提供抗原检测快速诊断测试的中心都配备了针对新型冠状病毒肺炎和结核病的快速分子检测平台、足够的实验室人员以及用于筛查的试剂和耗材。患者随访代理机构使用非书面结核病调查问卷，对到新型冠状病毒肺炎检测中心就诊的每个患者进行筛查。疑似结核病患者被要求提供痰液样本以进行快速分子检测。随后，我们改变了我们的操作流程，使用快速诊断测试对到结核病门诊就诊的患者也进行新型冠状病毒肺炎筛查。

**结果:**

2021 年 3 月至 12 月，我们对 14,588 名疑似新型冠状病毒肺炎患者进行了结核病筛查，其中 475 人 (3.3%) 被确认为患有疑似结核病。疑似结核病患者中有 288 人 (60.6%) 接受了检测，32 人 (11.1%) 被确认为结核病阳性（每 10 万人筛查中有 219 例病例）。在结核病阳性患者中，有 3 人患有利福平耐药结核病。在其余 187 个未经检测的疑似结核病患者病例中，174 人在随访时报告没有症状，13 人拒绝检测或无法跟踪。在对 671 名疑似结核病患者进行的新型冠状病毒肺炎筛查中，有 17 人 (2.5%) 通过抗原快速诊断检测呈阳性，有 5 人 (0.7%) 检测呈阴性，随后在分子检测平台上检测呈阳性（每 10 万人筛查中有 2,483 例新型冠状病毒肺炎病例）。

**结论:**

在印度进行新型冠状病毒肺炎和结核病同时筛查的举措是可行的，且可以改善新型冠状病毒肺炎和结核病的现场实时检测情况。

## Резюме

**Цель:**

Оценить внедрение новых рабочих процессов для одновременного проведения скрининг-тестов на коронавирусную инфекцию 2019 (COVID-19) и туберкулез в четырех крупных центрах тестирования на COVID-19, расположенных в специализированных больницах третьего звена в г. Мумбай, Индия.

**Методы:**

Каждый центр, в котором уже предлагалось проведение экспресс-диагностических тестов на выявление антигена, был оснащен платформой для быстрого молекулярного тестирования на COVID-19 и туберкулез, реактивами и расходными материалами для проведения скрининг-тестов, а также для каждого центра было предоставлено достаточное количество лабораторного персонала. Посредством устного опросника о состоянии здоровья при туберкулезе агент по последующему наблюдению за пациентами обследовал лиц, посещающих центры тестирования на COVID-19. Пациентов с подозрением на туберкулез просили предоставить образцы мокроты для проведения экспресс-молекулярной диагностики. Впоследствии рабочий процесс был изменен, и пациенты, посещающие туберкулезные амбулаторные отделения, также обследовались на наличие COVID-19 с использованием экспресс-диагностических тестов.

**Результаты:**

С марта по декабрь 2021 года на предмет наличия туберкулеза было обследовано 14 588 пациентов с предположительным диагнозом COVID-19, из которых у 475 (3,3%) был выявлен предположительный туберкулез. Из них 288 человек (60,6%) прошли тестирование, и у 32 человек (11,1%) был выявлен положительный результат на туберкулез (219 случаев на 100 000 обследованных). Среди больных туберкулезом у трех человек был обнаружен туберкулез, устойчивый к рифампицину. Из оставшихся необследованных 187 предполагаемых больных туберкулезом 174 человека сообщили об отсутствии симптомов при последующем наблюдении, а 13 человек отказались от обследования, либо их не удалось отследить. Из 671 случая предполагаемого туберкулеза, выявленного при скрининге на COVID-19, 17 (2,5%) оказались положительными при проведении экспресс-диагностики на антигены, а пять (0,7%) отрицательных тестов впоследствии оказались положительными при проведении молекулярного тестирования (2483 случая COVID-19 на 100 000 обследованных).

**Вывод:**

Одновременное проведение скрининг-тестов на COVID-19 и туберкулез в Индии является практически возможным и может способствовать выявлению COVID-19 и туберкулеза в исследовательском центре в режиме реального времени.

## Introduction

The overwhelming need for health care generated by the coronavirus disease 2019 (COVID-19) pandemic had a catastrophic effect on health-care programmes, particularly outpatient tuberculosis screening and detection. Before the COVID-19 pandemic, India already accounted for the highest number of tuberculosis cases in the world, with an estimated 2.95 million people falling ill annually.^1^ Because of rapid reductions in screening capacities for tuberculosis during the pandemic, there was an observable drop in the number of global tuberculosis case notifications: India alone contributed an estimated 41% to this decline in 2020.^2^ As a result, global tuberculosis-related deaths are expected to increase by about 20% over the next five years.^3^ The drop in notifications was linked to the diversion of health-care resources and personnel for COVID-19 response, and restrictions on movement during the pandemic, both of which prevented individuals from seeking regular, day-to-day preventive and primary health care. 

Throughout the pandemic, Indian officials encouraged the use of antigen-detecting rapid diagnostic tests as the diagnostic tool of choice for COVID-19, primarily because of their ease of use and rapid on-site results. As these rapid tests are sensitive but not highly specific, the Indian Council of Medical Research also mandated confirmatory testing using real-time reverse transcription polymerase chain reaction (RT–PCR) for all individuals negative on rapid diagnostic tests but presenting with distinctive COVID-19 symptoms.^4^ Under this guidance, health facilities offering COVID-19 confirmatory testing typically referred samples for RT–PCR to off-site council-approved laboratories. In many cases, this workflow led to long turnaround times and delays in the eventual diagnosis, isolation and management of patients.

Given the wide overlap in risk factors and symptoms in COVID-19 and tuberculosis, multiple stakeholders proposed simultaneous screening for COVID-19 and tuberculosis as a way to improve real time detection of these diseases, allowing for optimization of constrained health-care resources.^5^ In fact, India’s Ministry of Health and Family Welfare released a rapid response plan in 2020 recommending the simultaneous screening of COVID-19 and tuberculosis in both patient groups – i.e. all COVID-19 patients should be screened for tuberculosis and all tuberculosis patients should be screened for COVID-19.^6^ Further, officials from the Brihanmumbai Municipal Corporation in Mumbai issued guidelines reinforcing concurrent screening of patient attendees in outpatient tuberculosis and COVID-19 departments.

In consideration of the above challenges to diagnosis, we launched this study to facilitate a new operational model for the screening of simultaneous COVID-19 and tuberculosis at four high-volume tertiary hospitals in Mumbai, India. In collaboration with the Brihanmumbai Municipal Corporation, FIND and the Indian nongovernmental organization Confluence for Health Action and Transformation Foundation led the project. Here we describe the operational model these groups used to facilitate simultaneous screening programmes in Mumbai, and lessons learned which can be useful for implementation of simultaneous COVID-19 and tuberculosis screening approaches elsewhere, especially settings with a high burden of tuberculosis.

## Methods

### Study design

We designed this study as an implementation research study conducted at four high-volume COVID-19 rapid diagnostic testing centres in Mumbai from March to December 2021.

### Setting

Mumbai is one of the most populous cities in the world with 11.9 million inhabitants.^7^ The city has been especially hard hit by severe acute respiratory syndrome coronavirus 2 (SARS-CoV-2), with over 1.1 million COVID-19 cases and 19 500 deaths across Mumbai as of 2 May 2023.^8^ As such, officials in Mumbai have remained on high alert throughout the pandemic, focusing their efforts on containment through rapid diagnosis, isolation and appropriate clinical management.

The four tertiary hospitals selected offered walk-in COVID-19 testing using antigen-detecting rapid diagnostic tests, but also sent out samples for confirmatory molecular testing to referral laboratories. These hospitals primarily serve low-income residents of unregulated settlements (60–70%) and other mixed urban residents (30–40%).

### Recruitment of study participants

We included all people older than 18 years presenting themselves at either the COVID-19 testing centre or the tuberculosis outpatient department. To maintain uniformity in inclusion criteria, we excluded patients visiting other medical and general outpatient departments even if they subsequently tested positive for tuberculosis or COVID-19. All recruited participants were requested to provide verbal consent to participate in this screening. 

### Study implementation

We set up each study site in close proximity to the hospital’s COVID-19 rapid diagnostic testing centre to minimize potential challenges in sample collection and/or transportation of samples. We provided each centre with a Truenat™ (Molbio Diagnostics Pvt. Ltd, Verna, India) machine – a rapid molecular multidisease testing platform that uses RT–PCR to detect tuberculosis and COVID-19. Truenat™ has been endorsed by the World Health Organization, India’s National Tuberculosis Elimination Programme and the Indian Council of Medical Research for diagnosis of both tuberculosis and COVID-19.^9^^–^^11^ Noteworthy is that the study team allowed clinicians to use the Truenat™ platform to conduct in-house screening for COVID-19 on patients outside the study protocol.

We provided collaborating hospital laboratories with test chips, reagents and consumables for testing as well as one laboratory technician per laboratory. We recruited the laboratory staff using an advertised selection process and a technical interview with a FIND medical officer. A technical support team from Molbio Diagnostics trained all the recruited laboratory technicians and one technician from Brihanmumbai Municipal Corporation in rapid molecular sampling procedures. As required by the Indian government for all tuberculosis screening, the project team prepared a paper-based verbal questionnaire^12^ to screen for tuberculosis symptoms, in accordance with the National Tuberculosis Elimination Programme guidelines. Brihanmumbai Municipal Corporation subsequently approved the questionnaire.

Each site received a patient follow-up agent, who we trained at the onset of the project, via a 3-hour online orientation, on administering the questionnaire and the workflow of the project. Two months into implementation (May 2021), we provided a 3-hour online refresher training to the agents where they could ask questions regarding any difficulties they had experienced.

**Fig. 1:**
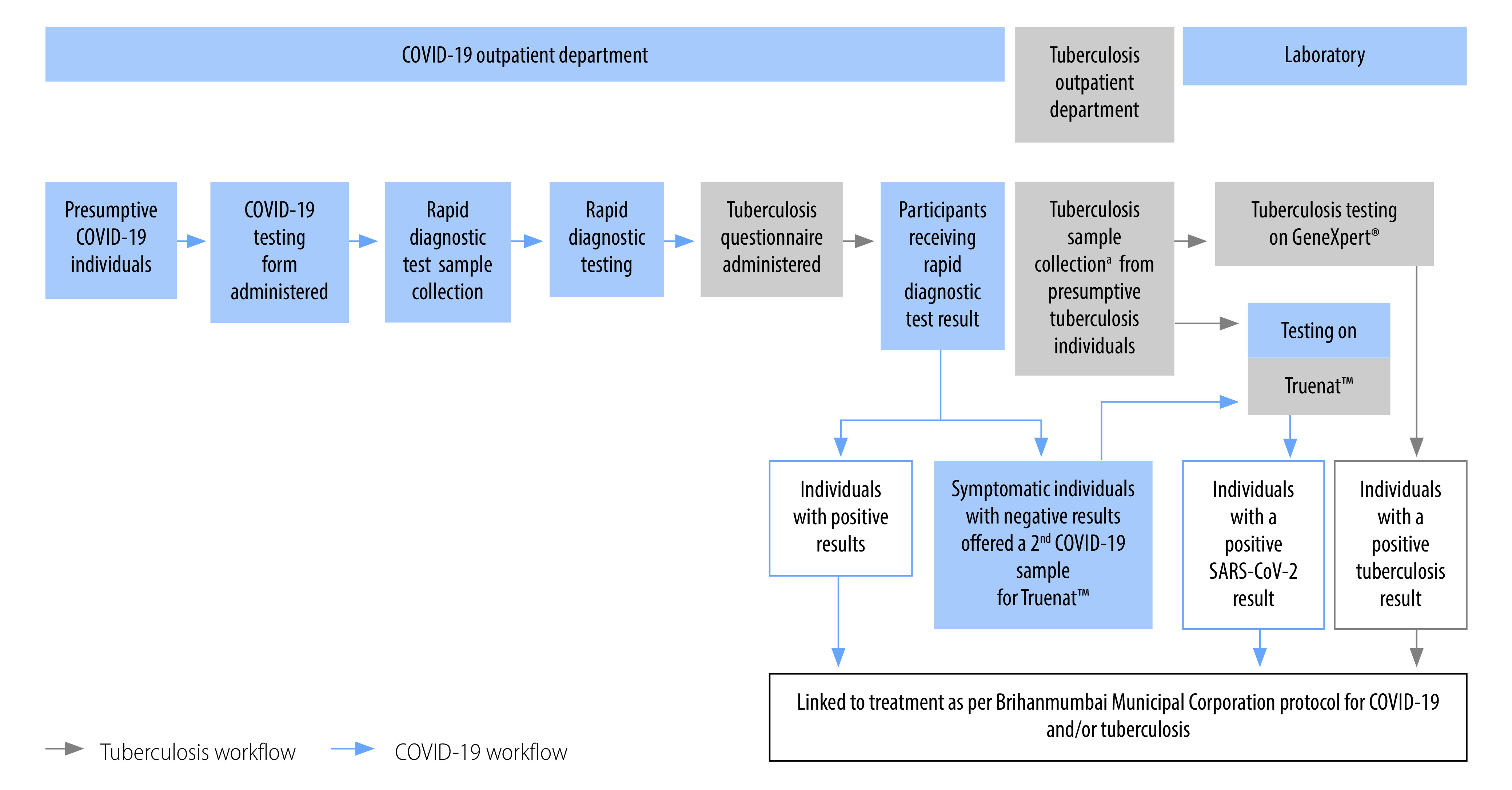
Flow of activities for COVID-19 and tuberculosis testing, India, 2021

COVID: coronavirus disease 2019; SARS-CoV-2: severe acute respiratory syndrome coronavirus 2.

^a^ Project staff phoned presumptive tuberculosis patients who had not provided a sputum sample within 14 days to enquire about symptoms and request return to the outpatient department in case of symptoms.

Note: For one site, the national tuberculosis elimination programme provided GeneXpert® for tuberculosis testing. A sample collection agent transported the samples to the laboratory.

Fig. 1 outlines how we screened presumptive COVID-19 cases for tuberculosis. Hospital staff registered individuals presenting at the COVID-19 outpatient department and collected samples for COVID-19 testing, before a patient follow-up agent screened them for tuberculosis by using the aforementioned screening questionnaire. Participants identified as presumptive tuberculosis cases on the verbal screening, irrespective of the COVID-19 result, had either their sputum sample collected on-site (one site had a collection area) or were asked to return the following day with a sputum sample (three sites did not have a collection area), for testing on the supplied Truenat™ machines. At one site, the National Tuberculosis Elimination Programme’s GeneXpert^®^ machine was also used for tuberculosis testing due to high demand for Truenat™ tests. Presumptive tuberculosis participants unable to produce a sputum sample were referred to a Brihanmumbai Municipal Corporation-run facility for follow-up and linkage to specialized diagnostic and treatment services. The project staff also phoned presumptive tuberculosis patients who had not provided a sputum sample within 14 days to enquire about symptoms, and request that individuals with symptoms return to the outpatient departments. All patients testing positive for tuberculosis were linked to Brihanmumbai Municipal Corporation for treatment and follow-up.

**Fig. 2:**
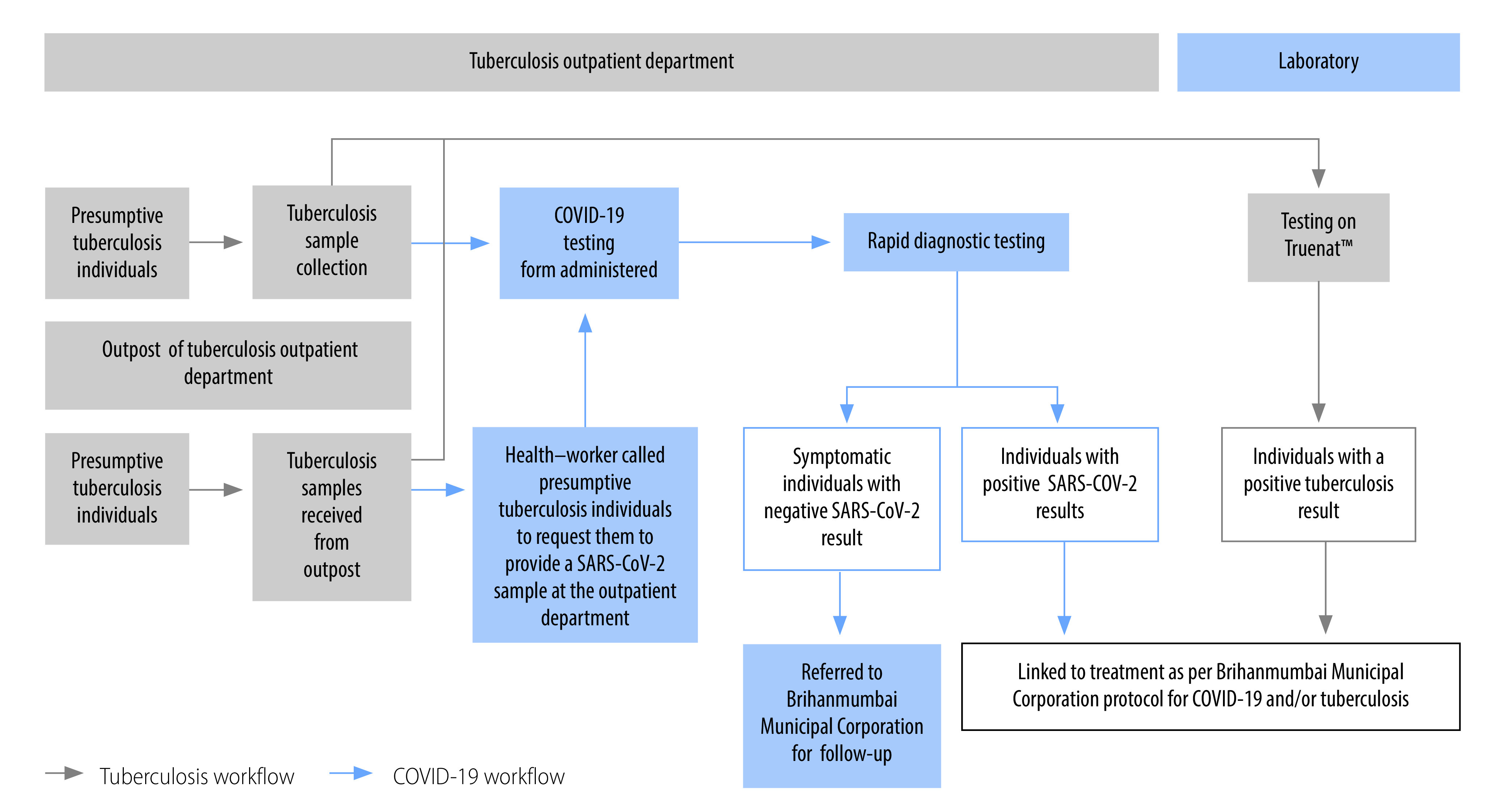
Flow of activities for COVID-19 and tuberculosis testing, India, 2021

COVID: coronavirus disease 2019; SARS-CoV-2: severe acute respiratory syndrome coronavirus 2.

Note: Samples were transported by a sample collection agent to the laboratory.

Beginning in mid-September 2021, we implemented a reversed operational workflow for simultaneous screening in three out of four sites via the process outlined in Fig. 2, largely in response to a steady decline in demand for COVID-19 rapid testing across all sites. While the earlier workflow screened presumptive COVID-19 cases for tuberculosis, the new workflow screened presumptive tuberculosis patients (walk-ins at the outpatient departments) for COVID-19 using the COVID-19 form and antigen-detecting rapid diagnostic tests. The workflow also included people whose tuberculosis samples were received from outposts linked to the outpatient department. Health workers phoned these patients and requested them to come into the outpatient department for COVID-19 rapid diagnostic testing. We introduced this workflow to reduce the substantial number of presumptive tuberculosis cases not screened for COVID-19 and to use excess Truenat™ capacity to reduce the number of sputum samples being sent outside the hospital for testing.

In this reversed workflow, the patient follow-up agent offered individuals visiting the tuberculosis outpatient departments COVID-19 testing. Subsequently Brihanmumbai Municipal Corporation personnel administered the antigen-detecting rapid diagnostic tests to those who accepted the offer. Consenting individuals who had symptoms of COVID-19 but tested negative by the rapid test had a second sample taken by a health-care worker for confirmatory rapid molecular testing on-site. All rapid molecular diagnostics performed with the Truenat™ platform for SARS-CoV-2 were conducted according to the Indian Council of Medical Research guidelines.^11^ Individuals with positive rapid and/or Truenat™ COVID-19 test results were referred to the Brihanmumbai Municipal Corporation programme for confirmation, including chest radiographs, and treatment. After three days, a member of the project team phoned asymptomatic individuals who tested negative on antigen-detecting rapid diagnostic tests to see if symptoms developed and, if so, asked them to return for further testing and contact tracing.

This study was regularly monitored via progress review meetings, site visits and joint meetings through a steering committee that consisted of Brihanmumbai Municipal Corporation, the Bill & Melinda Gates Foundation, Confluence for Health Action and Transformation Foundation and FIND.

### Cost of intervention

The project provided one laboratory technician and one patient follow-up agent per site, related stationery, personal protective equipment, as well as Truenat™ cartridges. While the patient follow-up agent was dedicated to administering the tuberculosis questionnaire, about 3% of the laboratory technician’s time was allocated to conducting tuberculosis tests at each site for the duration of the study. We used a time estimation analysis to determine the time laboratory technicians required for each task (Table 1). For both personnel, actual salaries were used to determine cost value.

**Table 1:** Data used for the time estimation of simultaneous screening for COVID-19 and tuberculosis, India, 2021

Variable	Test performed	
	COVID-19	Tuberculosis
No. of tests	15 000	288
Duration in minutes^a^	35	60
% time of laboratory technician spent	98.1	1.9
% of time considering duration of test	96.8	3.2
% used for calculation of time estimation	97.0	3.0

COVID-19: coronavirus disease 2019.

^a^ Excluding pre-processing time, which we assumed to be equal for both.

For the reversed screening conducted in tuberculosis outpatient departments, the project only provided a patient follow-up agent to each site, as the Brihanmumbai Municipal Corporation provided the COVID-19 rapid tests as an extension of a larger COVID-19 programme. Hence, the cost of the reversed screening could not be calculated.

### Variables and data sources

Key variables included in this study are: the total number of participants; the number of participants who tested positive for tuberculosis and/or COVID-19; and the percentage of participants who tested positive for tuberculosis and/or COVID-19. For tuberculosis-positive samples, we also recorded the presence of rifampicin-resistance. The project coordinator digitized the paper-based tuberculosis questionnaire used for screening individuals presenting for COVID-19 testing, in Microsoft Word (Microsoft Co., Redmond, United States of America) before sending the information to the Brihanmumbai Municipal Corporation staff. Using Microsoft Excel, we analysed all tuberculosis testing data, which on-site project personnel had collected using Brihanmumbai Municipal Corporation laboratory registers. Data on COVID-19 screenings in tuberculosis departments were collected using the Brihanmumbai Municipal Corporation’s COVID-19 tracker form in Excel.

Brihanmumbai Municipal Corporation staff cleaned all study data on-site and de-identified, meaning they only relayed anonymous patient data to the FIND team for final analysis. As the objective of the study was to screen and link patients for further evaluation per Brihanmumbai Municipal Corporation guidelines, de-identified individuals who tested positive under the intervention were reported via their case number to corporation teams at each testing site. Follow-up data on treatment or health outcomes post intervention were beyond the scope of this analysis.

### Ethical considerations

The study of simultaneous screening for COVID-19 and tuberculosis in Mumbai, India was approved as an intervention under the health department of Mumbai’s Brihanmumbai Municipal Corporation. We only initiated the study after receiving approval for collaboration from the Corporation. As simultaneous testing for COVID-19 and tuberculosis was recommended by the Indian Ministry of Health and Family Welfare and the Corporation as an intervention in all state hospitals, this study did not require specific ethical approval and was not conducted as a traditional research study, but rather as an evaluation of the implementation experience.

## Results

### Screening for tuberculosis

From March to December 2021, we screened a total of 14 588 presumptive COVID-19 patients for tuberculosis, and we identified 475 (3.3%) presumptive tuberculosis patients (Table 2). Of the presumptive tuberculosis patients, 286 (60.2%) were male and 188 (39.6%) were female; sex for one individual was unknown. Among the presumptive tuberculosis patients, 288 (60.6%) were tested for tuberculosis and 32 (11.1%) found to be tuberculosis positive (219 cases per 100 000 individuals screened), three of whom were further diagnosed with rifampicin-resistant tuberculosis. As for the 187 identified presumptive tuberculosis cases who were not tested, 174 reported no symptoms at 14 days follow-up and 13 individuals either refused testing or could not be traced.

**Table 2:** Participants screened at either COVID-19 testing sites or at tuberculosis outpatient departments, India, 2021

Outcome	No. of participants (%)	
	Screened at COVID-19 testing sites (*n* = 14 588)	Screened at tuberculosis outpatient departments (*n* = 886)
**Participants tested for COVID-19^a^**	8118 (55.6)^b^	671 (75.7)
**Confirmed COVID-19 cases**	523 (6.4)	17 (2.5)^c^
**Presumptive tuberculosis patients identified**	475 (3.3)	886
**Samples received for tuberculosis testing**	288 (60.6)	843 (95.1)
Tuberculosis detected	32 (11.1)	175 (20.8)
Rifampicin-resistant tuberculosis detected	3 (1.0)	3 (0.4)
COVID-19 and tuberculosis detected	0 (0.0)	1 (0.0)
**Samples not received for tuberculosis testing**	187 (39.4)	NA
Symptoms not found on follow- up	174 (93.0)	NA
Refused testing and follow-up	13 (7.0)	NA

COVID-19: coronavirus disease 2019; NA: not applicable.

^a^ Participants tested with a severe acute respiratory syndrome coronavirus 2 antigen rapid diagnostic test.

^b^ Only includes participants whose reports were available.

^c^ Not included are five cases that tested positive when using Truenat™ confirmatory testing. A total of 41 negative samples were retested.

Among the 14 588 individuals, rapid diagnostic tests were administered to 8118 (55.6%), and 523 (6.4%) were COVID-19 positive. Those not tested by rapid diagnostic tests may have received RT–PCR testing from the Brihanmumbai Municipal Corporation, and were not therefore included in participant follow-up exercises.

### Screening for COVID-19

Between September and December 2021, we offered screening to a total of 886 presumptive tuberculosis cases for COVID-19 (Table 2). Of these, 470 (53.0%) were male and 363 (41.0%) were female; sex was unknown for 53 individuals. Among the individuals screened for COVID-19, 671 (75.7%) were tested for COVID-19 using either antigen-detecting rapid diagnostic tests or Truenat™ machines. Only 17 individuals (2.5%) were identified as COVID-19 positive from the rapid tests. Additionally, 41 symptomatic individuals who tested negative via the rapid test permitted a second sample to be taken for confirmatory testing using rapid molecular methods. From this cohort, a further five individuals (12.2%) were identified as COVID-19 positive using the Truenat™ platform, resulting in average incidence of 2483 cases per 100 000 individuals screened over the project period.

Among the presumptive tuberculosis cases, 843 (95.1%) were tested for tuberculosis, with 175 (20.8%) identified as tuberculosis positive. Three tuberculosis-positive participants were diagnosed with rifampicin-resistant tuberculosis, and one participant was infected with both tuberculosis and COVID-19.

### Impact of the simultaneous screening intervention

In total, this study facilitated 1131 rapid molecular tuberculosis tests among presumptive COVID-19 cases, and diagnosed 207 individuals with tuberculosis, six of whom had rifampicin-resistant tuberculosis. The study also facilitated 712 rapid antigen and rapid molecular COVID-19 tests for presumptive tuberculosis patients, and subsequently diagnosed 22 presumptive tuberculosis patients with COVID-19.

### Cost

The total cost of simultaneous screening for COVID-19 and tuberculosis outpatient walk-ins between March and December 2021 was 19 792 United States dollars (US$) or US$ 1.4 per participant screened. This cost includes personnel, printing costs (for tuberculosis questionnaire forms), personal protective equipment and Truenat™ tuberculosis cartridges directly attributable to the study.

## Discussion

This study of simultaneous COVID-19 and tuberculosis screening in outpatient facilities at tertiary hospitals in India demonstrates that this operational model can be successfully implemented across health facilities using simple verbal screening and inclusion procedures and rapid molecular testing on-site. This study was conducted in close collaboration with the Brihanmumbai Municipal Corporation and the steering committee that closely monitored progress with follow-up visits to implementation sites, made review phone calls and timely course corrections. This approach ensured smooth operations for the duration of the study, as well as an effective handover of the study intervention to Brihanmumbai Municipal Corporation after completion. 

We observed that although simultaneous screening could be facilitated in either direction, COVID-19 screening in tuberculosis outpatient departments was more streamlined than screening for tuberculosis at COVID-19 testing sites. Reasons include: a verbal questionnaire was not required for tuberculosis patients being screened for COVID-19; tuberculosis clinics were less crowded than COVID-19 testing sites (fewer people walking in per day); and people presenting for tuberculosis testing were more open to being screened for co-morbid illnesses than those presenting for COVID-19 testing alone.

This study also demonstrates that the operational workflows for tuberculosis and COVID-19 screening in India can be harmoniously combined, aided by the fact that the same local government manages the COVID-19 and tuberculosis departments. We also noted that closed rapid molecular testing platforms such as Truenat™ are well suited for deployment in outpatient laboratories, as they require minimal infrastructure for set-up and deployment while providing an affordable end-to-end solution for sample collection and results reporting in just a few hours.^9^

Although the overall study and its implementation were successful, we encountered some barriers along the way. First, availability of sufficient personnel was a challenge, especially during COVID-19 surges, as there were high rates of infection and sick leave across deployed staff, despite staff being vaccinated and following appropriate infection control measures. The reduced number of personnel available potentially affected the number of people that were recruited for screening. Second, many participants identified as presumptive tuberculosis patients and who were asked to return with their sample at a later date did not return. Additionally, recruited participants were confined to those who self-triaged for COVID-19 and presented at the various tertiary hospitals included in this study, potentially biasing the overall sample.

We designed the project to be sustained beyond the initial implementation, with the project transitioned to Brihanmumbai Municipal Corporation staff after completion. However, we identified several factors as potential limitations towards the ongoing sustainability of the project. First, available personnel cross-trained to collect, analyse and report test results for more than one disease were lacking at the Brihanmumbai Municipal Corporation sites. To overcome this challenge, the study team provided Brihanmumbai Municipal Corporation with additional Truenat™ machines to test concurrently for COVID-19 and tuberculosis on-site. The study also built capacity among laboratory technicians for both testing protocols. A second limitation was the space constraints in hospitals, and the fear of patients releasing aerosols containing SARS-CoV-2 during sputum collection, which hindered us from setting up on-site sputum collection areas in three out of four hospitals. The Brihanmumbai Municipal Corporation and the study hospitals have noted the lessons from these challenges to better respond to future outbreaks.

As the COVID-19 pandemic has now slowed to a near-endemic state, the study test sites have reverted to their original tuberculosis-related testing, with the supplied Truenat™ machines being used by the tuberculosis programme under Brihanmumbai Municipal Corporation supervision.

While this article describes the implementation of simultaneous screening for COVID-19 and tuberculosis in tertiary hospitals in India, a similar approach has been used in several other countries. For example, in Kaduna State, Nigeria, the implementation of integrated community testing for tuberculosis and COVID-19 using a mobile diagnostic facility was found to be feasible and impactful.^13^ The Nigerian intervention increased the access of community members to testing for COVID-19, tuberculosis and other diseases, enabled timely turnaround of test results and referral for further management and treatment.^13^ Moreover, a report of 12 countries implementing simultaneous testing for COVID-19 and tuberculosis found that patients seeking COVID-19 testing could also be targeted for tuberculosis screening in areas where there is a known high burden of tuberculosis.^14^ This report, although showing successes, also identified several difficulties related to the facilitation of simultaneous screening, such as a lack of resources for on-site sputum sample collection and loss of participants to follow-up due to off-site sample collection.^14^

This study demonstrates that existing rapid molecular testing technology can be leveraged for future outbreak response, such as to provide flexible and temporary testing capacity in the event of an outbreak or pandemic. This study also highlights several measures that can potentially increase the feasibility and yield of future simultaneous screening efforts. These measures include ensuring adequate staffing levels; offering segregated on-site sputum collection areas; and more effective infection prevention and control measures for laboratory staff at sample collection sites. Additionally, simultaneous testing interventions can be streamlined in the laboratory by using a single sample type and test cartridge if possible, and offering dual patient screenings at a single touchpoint, instead of sequentially.

COVID-19 and tuberculosis screening in India is operationally feasible and may improve the detection of COVID-19 and tuberculosis, particularly in settings where is a known high burden of both diseases. The experience gleaned from this study suggests that although operational workflows for tuberculosis and COVID-19 can be combined, COVID-19 screening using antigen-detecting rapid diagnostic tests at tuberculosis outpatient departments may be more practical and better received than attempting to screen for tuberculosis at COVID-19 testing sites.
